# Treatment with aripiprazole and topiramate in an obese subject with borderline personality disorder, obsessive-compulsive symptoms and bulimia nervosa: a case report

**DOI:** 10.4076/1757-1626-2-7288

**Published:** 2009-07-23

**Authors:** Antonio Bruno, Deborah Riganello, Antonio Marino

**Affiliations:** Institute of Psychiatric Rehabilitation “Villa S. Agata”, Via Rimembranze36, 89131 Gallina, Reggio CalabriaItaly

## Abstract

**Introduction:**

Borderline personality disorder is a chronic mental disorder associated with severe psychosocial impairment and morbidity, greater usage of mental health resources, and a high mortality rate. Although there is no drug with an approved indication for this disorder, pharmacological treatment is a common practice based on the specific benefit of the drugs on the remission of the core symptoms of the disease.

**Case presentation:**

Authors reported the case of a 37-year-old obese woman with borderline personality disorder, obsessive-compulsive symptoms and bulimia nervosa treated with aripiprazole and topiramate. Co-administration of aripiprazole and topiramate produced a significant improvement of all psychopathological dimensions, obsessive-compulsive symptoms, and eating disorder.

**Conclusion:**

Co-administration of aripiprazole and topiramate could be a safe and effective long-term treatment for improving not only the symptoms of borderline personality disorder but also the associated health-related quality of life and interpersonal problems.

## Introduction

Borderline personality disorder (BPD) is condition difficult to treat [1] which is characterized by three main psychopathologic dimensions: impulsiveness, an unstable sense of self with difficulty in interpersonal relationships and affective instability [2,3].

Although psychotherapy has been the main therapeutic approach for the disorder, current guidelines state that pharmacotherapy is indicated to manage state symptoms and trait vulnerabilities. Long-term treatment is necessary and although there is no drug with an approved indication for BPD, many drugs have showed efficacy on borderline symptomatology, improving the course of the disorder and making possible to conduct psychotherapeutical work with more beneficial results. Among available drugs, apart from SSRIs, the novel antipsychotic olanzapine emerges as one of the most studied pharmacological agents for short-term management of BPD; its administration, although effective, is often associated with weight increase and, consequently, particular caution must be warranted with regard to potential complications in comorbid eating disorders in BPD populations [4].

Authors reported a case of BPD with obesity, obsessive-compulsive symptoms and bulimia nervosa treated with aripiprazole and topiramate; besides the efficacy on BPD symptomatology [5-9], the therapeutic choice was oriented considering that both substances had favourable tolerability profile and a low propensity to cause weight gain, dyslipidemia, glucose dysregulation and metabolic syndrome [10-11].

## Case presentation

The patient is a 37-year-old Caucasian-Italian woman, unmarried, of medium socio-cultural level; the symptomatological onset (tendency to isolation, deflected mood, somatizations) dated at the age of 16 years old. Notwithstanding those symptoms, she maintained an acceptable level of functioning until the age of 22 years old, when, in relation with her father’s illness that would have led him to death within ten years, she displayed a symptomatology characterized by social withdrawal, and behavioural and eating disorders. From the age of 22 years to the age of 35 years, she was treated for Major Depressive Disorder with atypical antipsychotics, antidepressants, benzodiazepines, and psychotherapeutic interventions. Treatments were only partially effective. When, for the persistent symptoms and for the onset of serious family conflicts, she was referred at our Unit, in September 2006, weighting 123 Kg (BMI = 45) and completely toothless, she suffered from arterial hypertension and type 2 diabetes mellitus (fasting phase glycaemia = 15.5 mmol/l). For the psychiatric and clinical conditions, she was assuming the following pharmacological therapy: risperidone 2 mg/day, sertraline 50 mg/day, delorazepam 1 mg × 2/day, enalapril 20 mg/day, metformine 1000 mg × 3/day.

**Figure 1.**
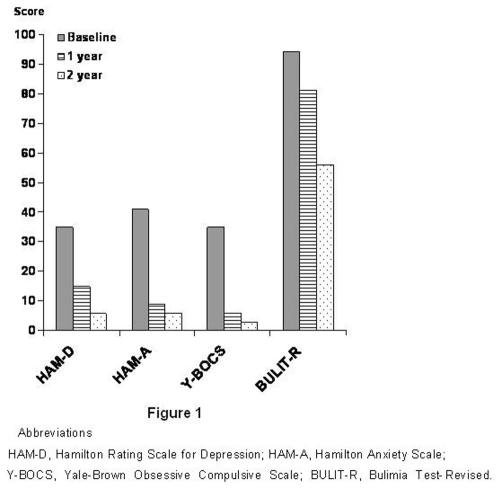
Total scores of rating scales at baseline and after 1 year and 2 years of aripiprazole plus topiramate treatment.

The clinical interview evidenced depressive mood, lack of self-esteem with explicit mistrust towards the possibility to be helped, a pervasive feeling of emptiness, fear of contamination with repeated hand washing, and intense anxiety. Regarding socio-relational functioning, her relationships were superficial and characterized by impulsivity and intense anger; towards her relatives, she constantly tended to ascribe them the responsibility and the guilt for her situation. Unable to recognize a link between her own emotions and life events, she immediately translated affective states in bodily correlates with a tendency to privilege a psychosomatic focus (the contact of her skin with fabrics different from silk caused her reddening and itch). Moreover, she exhibited a presumed intolerance towards smells and almost all the materials (wood, paper, steel). She also displayed an eating disorder marked by compulsive overeating without purging behaviours (Bulimic type). The patient underwent neurological examination with EEG, and allergologic visit with patch test which excluded any organic nature of the symptoms. The patient underwent a psychodiagnostic assessment which included: Borderline Personality Disorder Scale (BPD-Scale), Hamilton Rating Scale for Depression (HAM-D), Hamilton Anxiety Scale (HAM-A), Yale-Brown Obsessive Compulsive Scale (Y-BOCS), Bulimia Test-Revised (BULIT-R). The results of the assessment were as follow: BPD-scale = 40, HAM-D = 35, HAM-A = 41, Y-BOCS = 35, BULIT-R = 94 (Figure 1).

She was though diagnosed, according to DSM-IV criteria, as Borderline Personality Disorder with comorbid Obsessive-Compulsive Disorder and Bulimic Disorder; due to persistent symptoms and non-response to previous treatments, pharmacological treatment was accordingly modified by the progressive reduction until suspension of risperidone and sertraline and the introduction of aripiprazole 5 mg/day and topiramate up to 200 mg/day; the topiramate final dosage was reached within 4 weeks. Antihypertensive and hypoglycemic therapies were left unchanged; moreover, the patient engaged in supportive psychotherapy.

At one year follow-up, the combined treatment produced a significant improvement in mood and in socio-relational functioning; the patient also decided for the realization of a dental prosthesis. Gradually, obsessive thoughts and psychosomatic symptoms attenuated until disappear (she was able to wear whichever fabric); eating behaviour became more regular and the frequency of binge eating episodes decreased. Moreover, she began to collaborate with the cook in the preparation of meals and to give private lessons to the patients referred to our unit. A second psychodiagnostic assessment at the end of the first year of treatment evidenced the following results: HAM-D = 15, HAM-A = 9, Y-BOCS = 6, BULIT-R = 81 (Figure 1).

During the second year of treatment, a psychosomatic symptomatology related to the genital area emerged (reddening and itch). Considering the obtained results and the global functioning of the subject, it has been decided to leave unchanged pharmacological therapy and to continue psychotherapy with the aim to support the development of an independent self able to manage conflicts, the strengthening of ego functions, and the elaboration of the psychological abuse.

At two years follow-up, the improvement in psychopathology was furtherly maintained and consolidated; the patient went back home, her socio-relational functioning was marked by a greater degree of autonomy and a better control on emotional states and impulsivity. The final psychodiagnostic assessment at the end of the second year of treatment showed a further reduction of total scores: HAM-D = 6, HAM-A = 6, Y-BOCS = 3, BULIT-R = 56 (Figure 1).

Moreover, during the treatment a progressive loss of weight occurred (the patient actually weights 90 kg - BMI = 33) as well as the normalization of the glycaemic values (fasting phase glycaemia = 4.3 mmol/l) with the consequent discontinuation of the hypoglycaemic treatment; although antihypertensive therapy remained unchanged, blood pressure values were enough stable.

It can be observed that the 24-months treatment was effective in reducing depressed mood, anger outbreaks, anxiety, psychosomatic and obsessive symptoms, and, globally, socio-relational functioning.

## Discussion

Borderline personality, a pervasive disorder which has important clinical and social repercussions, has been mainly treated by psychotherapy. In recent years, the syndromic analysis of this disorder has allowed us to identify different symptoms susceptible of improvement with psychopharmacology treatment. Antidepressants, mood stabilizers, antipsychotics and benzodiazepines have shown efficacy in the treatment of symptomatic dimensions of the disorder. From a neurobiological point of view, a rationale exists for drugs acting on the dysfunction of those neurotransmitters that mediate behavioural responses and temperamental traits of vulnerability, mood, anxiety, and psychotic symptoms, and, finally, comorbid psychopathology. In particular, low levels of serotonine, cortical dopamine deficit and GABAergic deficit are considered as possible causes respectively for impulsivity, cognitive distortion, and emotional instability [4].

Aripiprazole is a novel antipsychotic, successfully employed for the treatment of schizophrenia and bipolar disorder, acting as partial agonist at dopamine D2 and serotonin 5-HT1A receptors and as antagonist at 5-HT2A [12]. Because of its ability to stabilize dopaminergic and serotonergic systems, aripiprazole may represent a new treatment approach for many other psychiatric disorders such as resistant depression, anxiety, obsessive compulsive disorder and BPD [13]. Aripiprazole exhibited a favorable safety and tolerability profile, with a low propensity to cause extrapyramidal symptoms, weight gain, cardiovascular abnormalities, hyperprolactinemia, hypercholesterolemia, or glucose dysregulation; however in phase 3 of the CATIE schizophrenia trial, unlike previous findings, it was associated with greater increases in blood glucose than all of the other treatment regimens [14].

Topiramate is an anticonvulsant and its multifactorial action mechanisms include blockade of voltage-dependent sodium channels, potentiation of GABAergic transmission and inhibition of excitatory pathways through an action at AMPA receptor sites [15]. Topiramate has shown a documented effect on the reductions of impulsive behaviours, such as alcohol and cocaine use, binge eating and purge behaviours in bulimia nervosa, and aggression in borderline personality disorder [16].

Based on this evidence, the association aripiprazole plus topiramate may represent a promising treatment for BPD and concurrent psychiatric disorders.

The clinical case reported has evidenced that previous treatments with atypical antipsychotics, SSRI and benzodiazepine did not resulted effective in improving the exhibited symptomatology; concurrently, the onset of significant metabolic problems (pronounced weight increase, hyperglycaemia) was observed.

Contrarily, the co-administration of aripiprazole plus topiramate induced a significant and longlasting improvement both of overall symptomathology, that is, the BPD core symptoms and comorbid conditions (obsessive symptoms, somatizations and eating disorder); a significant improvement of dysmetabolic aspects (weight loss, normalization of glycemic levels) also occurred. Result were coherent with previous studies demonstrating the efficacy and tolerability of both aripiprazole and topiramate in long-term treatment of BPD [5-11]; efficacy that is showed by the improvement of core symptoms of the disorder and, moreover, of quality of life and of socio-relational dysfunctions in borderline patients.

## Conclusions

Aripiprazole and topiramate co-administration could be a safe and effective long-term treatment for improving symptoms of Borderline Personality Disorder. To the best of our knowledge, scientific literature is actually lacking of controlled trials aimed to test the efficacy and the tolerability of the combination topiramate plus aripiprazole for the treatment of major psychiatric disorders, and of personality disorders, including BPD. Starting from the clinical experience presented in this case report, it would be therefore interesting to design controlled studies with the aim of evaluating the combination of aripiprazole plus topiramate in selected populations of psychiatric patients.

## Abbreviations

AMPA: a-amino-3-hydroxy-5-methyl-4-isoxazole propionic acid
BMI: Body Mass Index
BPD: Borderline Personality Disorder
BULIT-R: Bulimia Test-Revised
GABA: Gamma-aminobutyric acid
HAM-D: Hamilton Rating Scale for Depression
HAM-A: Hamilton Anxiety Scale
SSRI: selective serotonin reuptake inhibitors
Y-BOCS: Yale-Brown Obsessive Compulsive Scale
